# Hepatic Hydatid Cyst Complicated by Biliary Obstruction: A Case Report

**DOI:** 10.7759/cureus.85319

**Published:** 2025-06-04

**Authors:** Usamah Al-Anbagi, Abdelrahman Mostafa, Imran H Mohammed, Abdulqadir J Nashwan

**Affiliations:** 1 Internal Medicine Department, Hazm Mebaireek General Hospital/Hamad Medical Corporation, Doha, QAT; 2 Internal Medicine Department, Hamad Medical Corporation, Doha, QAT; 3 Nursing & Midwifery Research Department, Hamad Medical Corporation, Doha, QAT

**Keywords:** albendazole, biliary obstruction, echinococcus granulosus (e. granulosus), endoscopic retrograde cholangiopancreatography (ercp), hydatid cyst

## Abstract

A 35-year-old male, previously healthy, presented with epigastric and right hypochondrial pain, as well as a change in urine color. The diagnosis of a hepatic hydatid cyst with complications was confirmed based on clinical findings, ultrasound, and serology. The patient was treated with albendazole and underwent an ERCP (endoscopic retrograde cholangiopancreatography) for biliary involvement. Hydatid cysts are caused by *Echinococcus granulosus*, transmitted through contact with infected animals or contaminated food. The disease can lead to severe complications such as biliary obstruction, jaundice, and anaphylaxis. Early diagnosis through imaging and serology is essential, while treatment often involves a combination of antiparasitic therapy and surgical or percutaneous procedures. Long-term monitoring is required to detect recurrence, which can occur years after treatment.

## Introduction

Echinococcosis, or hydatid disease, is a parasitic infection caused by *Echinococcus granulosus* and *Echinococcus multilocularis* [1]. Humans are accidental hosts in the tapeworm's life cycle, with infection typically occurring through ingesting eggs from contaminated food, water, or contact with infected animals [1]. Although *E. granulosus* primarily affects the liver and lungs, it can also involve other organs such as the brain, kidneys, and heart [2]. The disease presents in various ways, often depending on the size and location of the cyst [2]. Treatment includes antiparasitic drugs such as albendazole, combined with surgical or percutaneous interventions for more complex cases [2]. Cystic echinococcosis remains a major public health concern in several endemic regions, with high prevalence rates in rural areas of South America, Central Asia, and parts of Africa and China [3]. Improved diagnostics have increased case detection, revealing notable infection rates in humans and livestock [3]. Strain variations of *E. granulosus* may impact transmission dynamics and influence control strategies in affected regions [3]. This case highlights a hepatic hydatid cyst with biliary involvement, emphasizing the importance of early diagnosis and management.

## Case presentation

A 35-year-old male, previously healthy with no comorbidities, presented with a three-day history of epigastric and right hypochondrial pain. The pain was moderate in intensity (5/10), non-radiating, and had no clear aggravating or relieving factors. He also reported a one-day history of a change in urine color to deep yellow. He denied any history of jaundice, mouth ulcers, dysphagia, dyspepsia, or bleeding from any orifice. He consumes fresh vegetables regularly, ensuring that they are well-washed. The patient denied fever, cough, shortness of breath, or other respiratory symptoms. He had no history of headaches, seizures, or other neurological symptoms and denied any direct contact with cattle, sheep, or dogs. There was also no family history of hydatid disease or liver disease.

The patient appeared well on physical examination, was fully conscious and oriented, and was hemodynamically stable. His temperature was 36.5°C, heart rate was 70 bpm, respiratory rate was 19 breaths per minute, blood pressure was 121/76 mmHg, and SpO2 was 98% on room air. He was not jaundiced or pale, was adequately hydrated, and had no lymphadenopathy or lower limb edema. On abdominal examination, there was tenderness over the upper abdominal regions, and the liver was palpable, measuring approximately 16 cm in span with a nodular surface. Bowel sounds were normal. The remaining clinical examination was unremarkable. Laboratory investigations revealed slightly elevated inflammatory markers and transaminitis. A positive titer for *E. granulosus *antibodies was found (Table 1).

**Table 1:** Laboratory investigations ALT, alanine aminotransferase; AST, aspartate aminotransferase; CRP, C-reactive protein; PT, prothrombin time; INR, international normalized ratio; APTT, activated partial thromboplastin time

Parameters	On admission	On discharge	Reference values
Total leukocytes (x10^3^/uL)	10.2	23	4.0-10.0
Hematocrit (%)	45	43	40-50
Hemoglobin (gm/dL)	15.5	14.6	13-17
Platelet (x10^3^/uL)	232	236	150-410
Serum urea (mmol/L)	2.1	4.6	2.5-7.8
Serum creatinine (umol/L)	60	71	62-106
Serum potassium K (mmol/L)	3.6	4.2	3.5-5.3
Serum sodium (mmol/L)	138	137	133-146
Serum calcium (mmol/L)	2.34	-	2.2-2.6
Serum total protein (gm/L)	82	77	60-80
Serum albumin (gm/L)	38	30	35-50
ALT (IU/L)	414	148	0-41
AST (IU/L)	114	57	0-41
Alkaline phosphatase (U/L)	273	424	40–129
Serum total bilirubin (mg/dL)	55	39	0-21
CRP (mg/L)	11	27	0-5
Procalcitonin (ng/mL)	-	0.17	<0.05
PT (seconds)	13.4	16.3	9.4-12.5
INR	1.2	1.4	<1
APTT (seconds)	37.4	41.2	25.1-36.5

**Figure 1:**
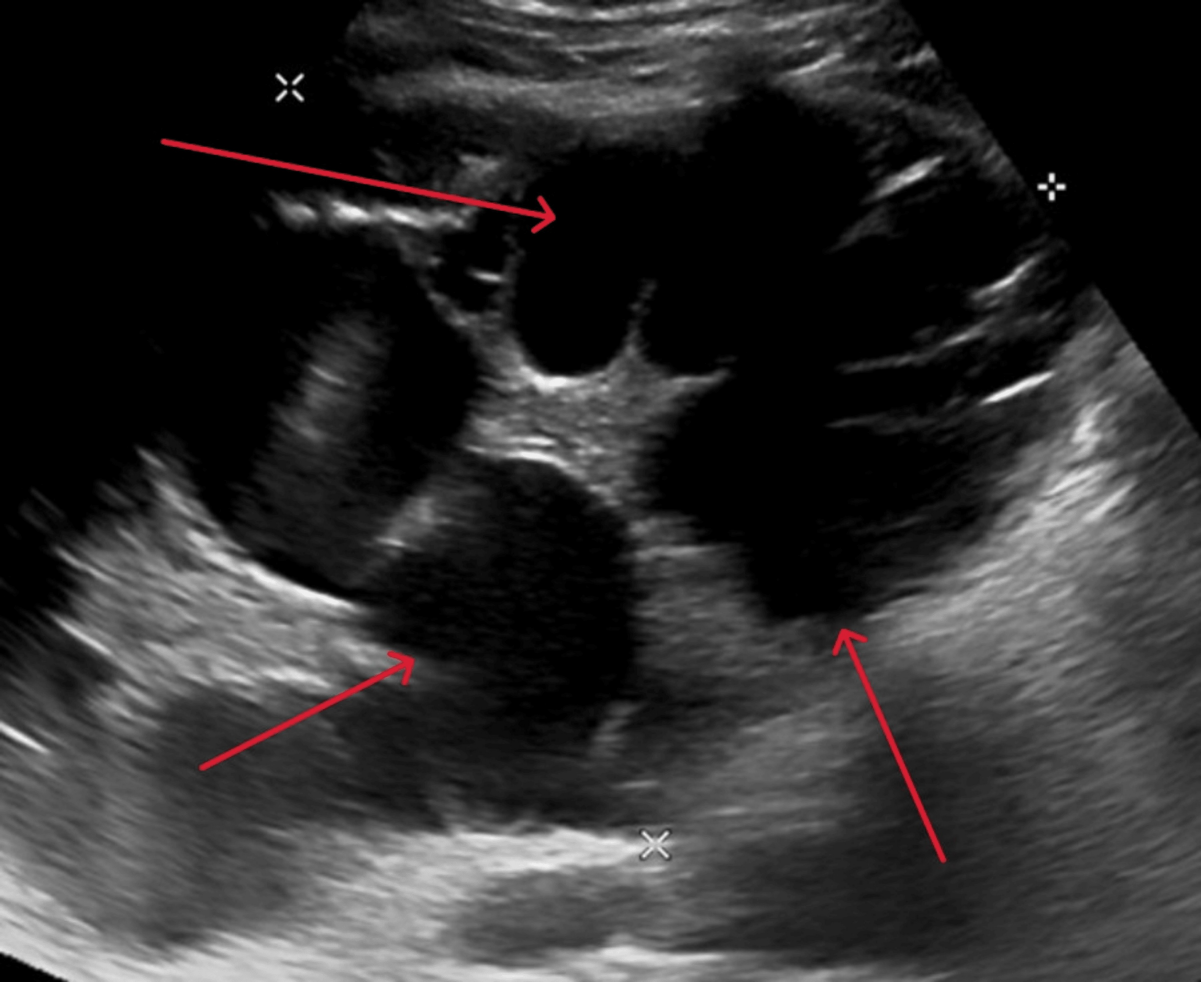
Multiloculated cystic lesion in the right lobe of the liver Abdominal ultrasound showing mild hepatomegaly (liver span: 15.5 cm) and a large multiloculated cystic lesion measuring 16 × 12 cm located in the right hepatic lobe (highlighted by red arrows).

**Figure 2:**
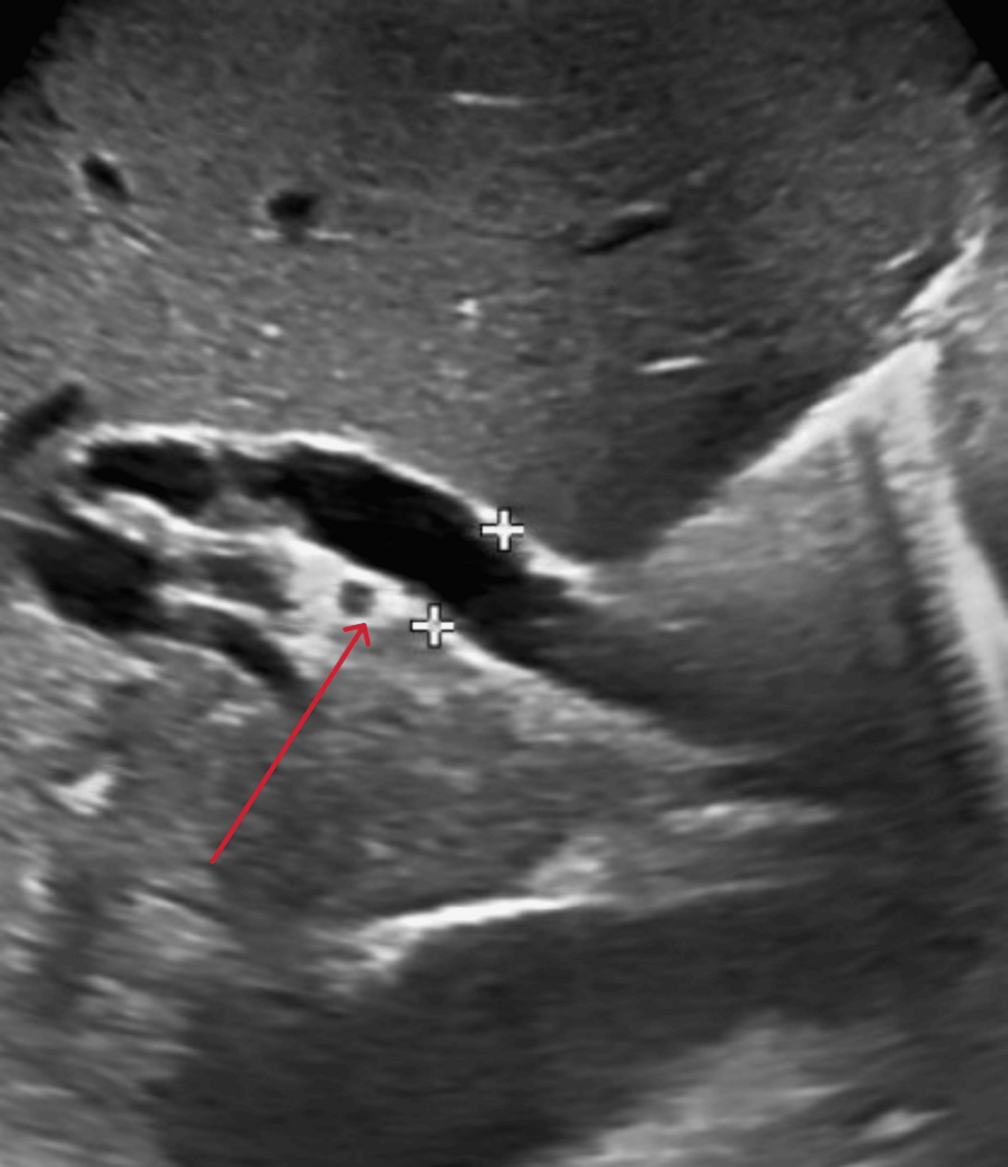
Abdominal ultrasound showing dilated common bile duct Abdominal ultrasound image showing a dilated common bile duct (red arrow), suggestive of possible biliary obstruction or underlying pathology requiring further evaluation.

An abdominal ultrasound showed mild hepatomegaly (15.5 cm in span) and a 16x12 cm multiloculated cystic lesion in the right lobe of the liver (Figure 1), with a dilated common bile duct measuring around 14 mm (Figure 2).

**Figure 3:**
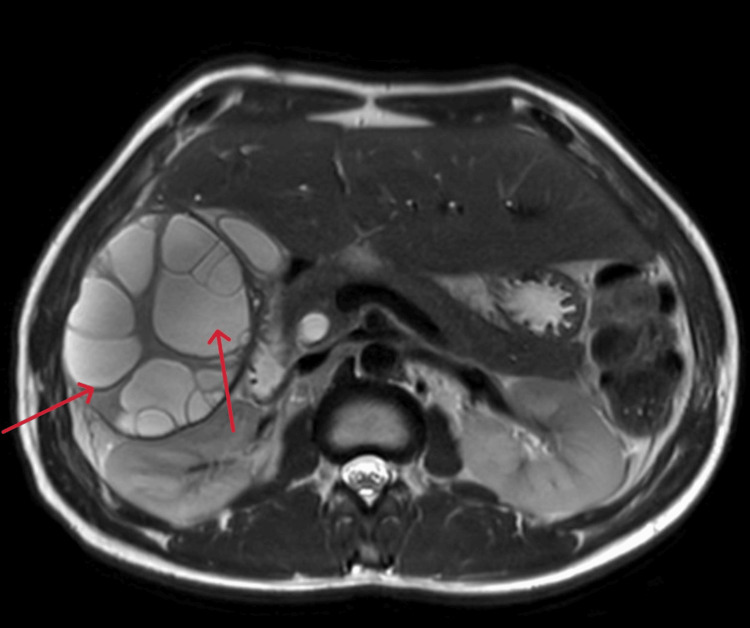
Axial MRI of the liver demonstrating hydatid cyst Axial MRI image of the liver showing a large hydatid cyst involving the right hepatic lobe. Multiple peripherally arranged daughter cysts are clearly visible within the main cystic lesion. MRI, magnetic resonance imaging

**Figure 4:**
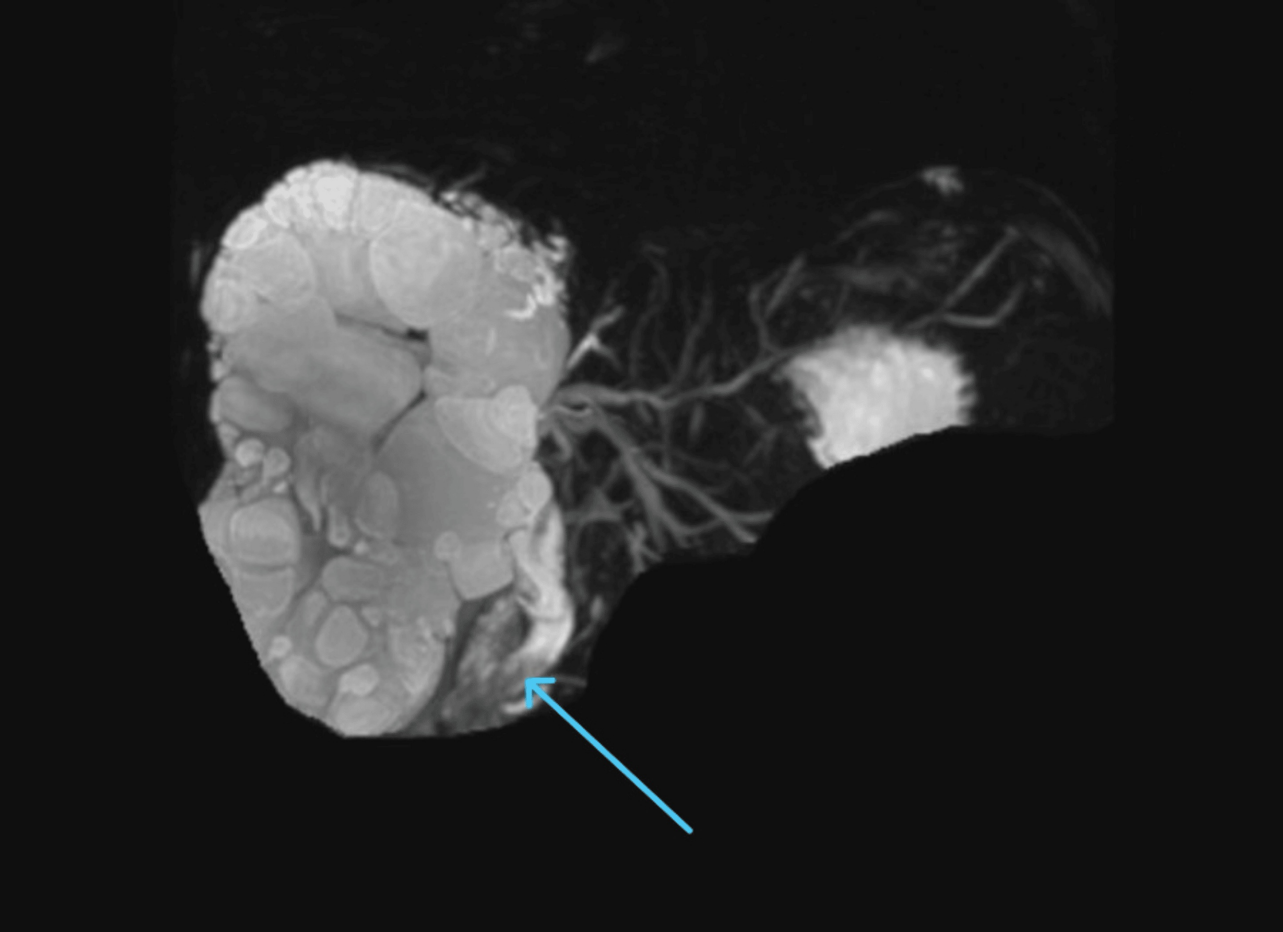
MRCP revealing common bile duct dilatation with intraluminal debris MRCP image showing dilatation of the common bile duct with visible intraluminal debris (blue arrow), indicative of possible biliary obstruction or cholangitis. MRCP, magnetic resonance cholangiopancreatography

The patient was admitted with a suspected hepatic hydatid cyst, which was confirmed by radiological and laboratory findings. A magnetic resonance cholangiopancreatography (MRCP) was performed, which revealed a right hepatic lobe hydatid cyst with biliary communication and biliary obstruction (Figures 3, 4).

The patient was started on albendazole 400 mg twice daily, along with empirical metronidazole and cefuroxime, which were discontinued after a few days. The patient underwent an ERCP (endoscopic retrograde cholangiopancreatography) on day 6, which included biliary sphincterotomy and partial extraction of cyst membranes. A bile duct stent was placed. The patient was discharged in stable condition with follow-up appointments scheduled with a gastroenterologist and an infectious disease specialist. He was prescribed albendazole 400 mg twice daily for a planned treatment duration of three to six months. The patient was seen in the clinic two weeks later and was doing well with significantly improved symptoms.

## Discussion

Echinococcal disease is caused by infection with the *Echinococcus* tapeworm, primarily *E. granulosus* and *E. multilocularis*, which cause cystic and alveolar echinococcosis, respectively [1]. The life cycle involves definitive hosts (dogs or canids) and intermediate hosts (sheep, goats, camels, or cattle), while humans are incidental hosts [1]. Adult tapeworms in definitive hosts produce eggs excreted in feces, which remain viable for months. Ingestion of these eggs by humans leads to the formation of hydatid cysts, primarily in the liver [1]. Transmission occurs via fecal-oral contamination through contact with infected dogs, contaminated water, or unwashed vegetables [2]. The disease is most prevalent in sheep-raising regions, and human-to-human transmission does not occur [2].

The hydatid cyst, typically fluid-filled, consists of an inner germinative layer that produces hydatid fluid and secondary cysts (brood capsules), which can further develop into daughter cysts. Over 10 to 12 months, protoscolices form within the brood capsules, making the cyst fertile and capable of generating daughter cysts, while sterile cysts lack protoscolices. Surrounding the germinative layer is a laminated acellular membrane, encased by a host-induced granulomatous reaction known as the pericyst. The characteristics of *E. granulosus* cysts vary by region, with large, fertile cysts more common in Turkana, Kenya, while smaller, calcified, infertile cysts are seen in the northern hemisphere [4]. This variation may result from differences in infection duration, parasite strains, and host factors such as immunity, genetics, or nutrition [1]. The host mounts both humoral and cellular immune responses, initially against the oncospheres and later against the metacestode (hydatid cyst). However, the parasite employs immune evasion mechanisms, including protective membranes and immunomodulatory substances, such as an anticomplement factor [5].

The clinical presentation of *E. granulosus* infection depends on cyst size and location. Small or calcified cysts may remain asymptomatic, but larger cysts can cause symptoms from mass effect, obstruction, rupture, or infections. Cysts grow 1-5 cm yearly, with variable rates [6]. The liver is most commonly affected, followed by the lungs, and other organs such as the brain, kidneys, and heart are less often involved. Single-organ involvement occurs in 85-90% of cases, with over 70% having only one cyst. *Echinococcus granulosus* infection of the liver often presents with no symptoms, and the right lobe is affected in 60-85% of cases. Significant symptoms usually occur only when cysts reach 10 cm in diameter, leading to hepatomegaly, right upper quadrant pain, nausea, and vomiting. In around 25% of cases, cysts rupture into the biliary tree, causing biliary colic, jaundice, cholangitis, or pancreatitis [7-8]. Rupture can also lead to complications such as cholestasis, portal hypertension, and Budd-Chiari syndrome. Additionally, liver cyst rupture into the peritoneum may cause peritonitis, anaphylaxis, or dissemination of protoscolices. Secondary bacterial infections can result in liver abscesses, and rupture into the pleural space can cause pulmonary hydatidosis or a bronchial fistula.

*Echinococcus granulosus* infection may present with nonspecific lab findings, such as leukopenia, thrombocytopenia, mild eosinophilia, and liver function abnormalities, but these are not diagnostic. Eosinophilia occurs in fewer than 15% of cases, usually when antigenic material leaks from cysts. For asymptomatic individuals in endemic areas, screening with imaging and serology is valuable for early detection, as demonstrated in epidemiologic studies [9]. Diagnosis is primarily made through imaging techniques, including ultrasonography, computed tomography (CT), and magnetic resonance imaging (MRI), alongside serology. Ultrasonography is commonly used for screening and can identify cysts with characteristics such as hydatid sand, daughter cysts, and the "water lily sign." CT is more sensitive (95-100%) for locating cysts [10-11], detecting extrahepatic cysts, and assessing complications such as infection or intrabiliary rupture. Serology, particularly antibody detection, is crucial for diagnosis and follow-up, while PCR is still in research [12]. If serology is inconclusive, percutaneous aspiration or biopsy may be required to identify protoscolices or hydatid membranes, though this procedure carries risks of anaphylaxis and secondary infection spread [13]. ERCP may be necessary to evaluate biliary involvement in cases of cholestatic jaundice, revealing hydatid membranes or filling defects in the biliary tree.

The treatment of echinococcosis generally involves antiparasitic therapy, often combined with either surgical resection or percutaneous aspiration with scolicidal agents [13]. Albendazole is the primary treatment, taken with food to enhance absorption. For small cysts (WHO stage CE1 and CE3a, <5 cm) [9], drug treatment alone may be sufficient, with a duration of one to three months, though it can be extended [9]. Larger cysts or more complex stages (CE2 and CE3b) usually require surgery or percutaneous management [9]. Surgery is preferred for complicated cysts (ruptured, infected, or compressing vital structures) or large cysts (>10 cm) [14]. Laparoscopic surgery may be used for anteriorly located hepatic cysts, though it carries a risk of spillage due to increased intraabdominal pressure [15]. Percutaneous management, including the PAIR technique, is effective for cysts without daughter cysts (CE1, CE3a), while drainage with a large-bore catheter may be needed for cysts with daughter cysts (CE2, CE3b) [16]. For biliary complications, ERCP is often required [17]. Procedures such as sphincterotomy, bile duct extraction, and stenting may be needed for managing obstructive jaundice, cholangitis, or biliary fistulas, and multiple ERCP sessions may be necessary for stricture resolution [17].

Cystic echinococcosis can relapse years after treatment, with the natural history of the infection being highly variable. Cysts may grow, remain stable, rupture, or resolve spontaneously. Monitoring treatment success is challenging, and the approach should be individualized based on patient factors and available resources. Typically, follow-up includes imaging, such as ultrasound, CT, or MRI, every three to six months until stability is achieved. This is followed by annual monitoring, often for up to five years, to check for recurrence [18]. Ultrasonography can assess treatment response, showing signs such as cyst disappearance, size reduction, and thickening of the cyst wall, while new cysts or increased cyst size may suggest relapse. Antibody titers typically decrease after successful treatment, but some may remain elevated for years. The outcome depends on the stage of the disease, with many asymptomatic patients remaining stable over time. Cyst calcification, which takes 5 to 10 years to develop, particularly in hepatic cysts, may indicate cyst nonviability.

## Conclusions

This case highlights the importance of early recognition of biliary complications in hepatic hydatid disease, particularly in endemic areas. The patient’s right upper quadrant pain and jaundice prompted timely imaging, which confirmed a hepatic hydatid cyst with biliary involvement. Cross-sectional imaging played a crucial role in diagnosis and guided appropriate therapeutic planning, including using albendazole and ERCP. Long-term postoperative monitoring with imaging and serology is essential due to the risk of late recurrence. While this case illustrates a successful outcome, conclusions should be drawn with caution, as it reflects the experience of a single patient.
